# Application of Patient-Generated Health Data in Managing Chronic Conditions in Hospital Kuala Lumpur: A Qualitative Study

**DOI:** 10.21315/mjms2022.29.3.10

**Published:** 2022-06-28

**Authors:** Ao Lik Lee, Nik Nur Eliza Mohamed

**Affiliations:** 1 School of Medicine, Trinity College Dublin, College Green, Dublin 2, Republic of Ireland; 2 Clinical Research Centre, Hospital Kuala Lumpur, Kuala Lumpur, Malaysia

**Keywords:** patient-generated health data, patient-generated clinical data, patient-generated data, self-recorded health, medical informatics

## Abstract

**Background:**

Patient-generated health data (PGHD) is health-related data captured and recorded by patients which informs healthcare practitioners (HCP) about the patients’ health status between clinic visits. PGHD could be attributed as part of digital health and technological advancement.

**Methods:**

This is an exploratory qualitative study to explore the current PGHD usage and the views and experience of HCP towards PGHD. Semi-structured in-depth online interviews based on the modified Unified Theory of Acceptance and Use of Technology (UTAUT) were conducted with seven Hospital Kuala Lumpur medical- and surgical-based HCP specialists between October 2019 and February 2020. Purposive sampling method was applied to ensure speciality diversity among study respondents. Thematic analysis was performed on the interview transcripts.

**Results:**

Four main themes were identified namely the PGHD usage among the study respondents, the benefits of PGHD, the challenges of PGHD usage and the effort needed to use the PGHD. The main finding of this study includes the exploration of the benefits of PGHD usage such as efficient data management in aiding clinical consultation. Nonetheless, the clinical decision making based on PGHD is limited due to poor adoption of PGHD and unavailability of electronic data. This could be due to the lack of awareness, ICT infrastructure, funding, poor health literacy and language and cultural problems.

**Conclusion:**

PGHD has huge potential to be adopted in the clinical setting and subsequently benefiting the patients. However, parallel supportive environment is essential in supporting the usage of PGHD in the clinical setting.

## Introduction

Patient-generated health data (PGHD) is defined as health-related data created, recorded, gathered or inferred by patients or their designees to help address a health concern (1). Examples of PGHD include medical history, medications list and adherence, patient reported outcome (PRO), audio (e.g. cognitive behavioural therapy diary), images (e.g. skin condition), lifestyle (e.g. diet, exercise, smoking and alcohol consumption) and biometrics (e.g. weight, blood pressure, heart rate, temperature and glucose level) (1–3). PGHD is different from most data generated in healthcare settings in which patients, instead of healthcare practitioners (HCP), are responsible for recording data outside clinic visits (1). In Malaysia, PGHD can be roughly divided into three main types: i) quantitative; ii) qualitative and iii) multimedia, as shown in Table 1.

The awareness of PGHD is related to the health literacy of the population. Health literacy is defined as the degree to which individuals have the capacity to obtain, process and understand basic health information and services needed to make appropriate health decisions (4). The Malaysia National Health and Morbidity Survey 2015 report found that only 6.6% of the population has adequate health literacy while 93.4% has limited health literacy (4). This figure is extremely poor as compared to the US National Assessment of Adult Literacy in 2003, where 36% had limited health literacy (5).

Mobile Health (mHealth) is the application of mobile technologies, e.g. smartphones, tablets, wearable devices and sensor technologies, for the purpose of healthcare (6). A US study in 2016 showed that 46% of consumers have used three or more digital health tools and 25% owned wearable health-trackers (2). Lai et al. (6) found that 78% are willing to wear health-tracking technology, while 10% use it to manage chronic conditions. Comparatively, most mHealth studies in low-middle income countries including Malaysia primarily focus on using text messaging for behavioural change and only a few examined how mHealth could strengthen the health system (7). In a survey conducted in Selangor, only 20% of respondents were familiar with the term ‘mHealth’ or had used one; the most common being adherence aids or glucose meters apps (8). However, mHealth in Malaysia has added a new page since the introduction of MySejahtera to help in contact tracing of COVID-19 patients. It has reached over 23 million registered users including their dependents and 1 million registered business and premises as on 18 November 2020 (9).

**Figure 1:**
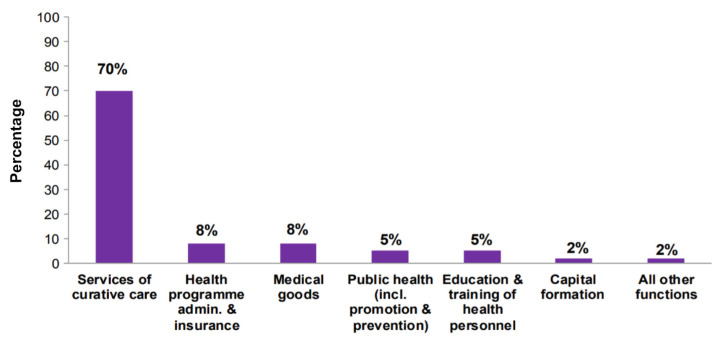
Total health expenditure on health by functions of healthcare

The Ministry of Health has been working to upgrade Information and Communication Technology (ICT) infrastructure in healthcare (10). They developed an electronic medical record (EMR) (*Sistem pengurusan pesakit*) and launched an online portal known as myHealth (11). However, the use of electronic PGHD is still limited as most medical records in Malaysia are paper-based due to lack of funding. As of 2019, only 20% of hospitals have EMR (12). According to health expenditure report, only 2.4% of funds is spent on capital formation (Figure 1), of which only 5% of capital expenditure was spent on ICT infrastructure (10, 13).

Most of the previous research regarding PGHD had explored the perception of patients and clinicians using semi-structured interview approach (6). Seto et al. (14) explored the experience of heart failure patients using mobile phone-based telemonitoring system to record their daily weight, blood pressure, weekly echocardiogram and symptoms questionnaire. Cohen et al. (15) explored the HCP’s perspective towards integrating PGHD into clinical care setting and concluded that PGHD provides value to both patients and HCP.

The purpose of remote patient monitoring is to effectively integrate data into clinical workflow (16). However, there is a lack of PGHD research in the context of Malaysia. To address this research gap, the present study aims to explore the experience of HCP using PGHD in Hospital Kuala Lumpur (HKL) in order to recommend PGHD incorporation into the current clinical workflow.

## Methods

### Study Design and Study Tool

**Figure 2:**
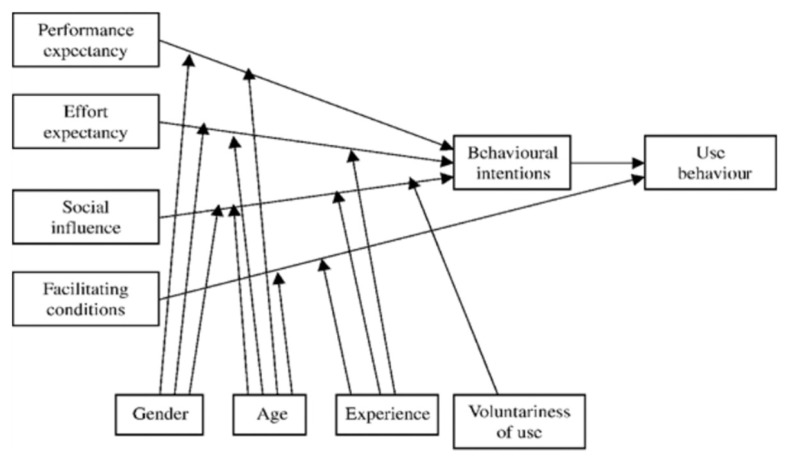
UTAUT model by Venkatesh et al. (17)

This is an exploratory qualitative study on the usage of PGHD in managing chronic patients using online semi-structured in-depth interviews. The interview questions were based on the modified Unified Theory of Acceptance and Use of Technology (UTAUT) (Figure 2), a conceptual model consisting of four key constructs: i) performance expectancy; ii) effort expectancy; iii) social influence and iv) facilitating conditions (17). In this study, the exploration on HCP experience utilising PGHD were focused on specific themes, namely PGHD use in Malaysia, benefits, barriers and effort needed to use PGHD.

### Study Population and Sampling Method

The inclusion criteria for the study participants were HKL specialists who manage patients with chronic conditions and are able to attend online interviews and had given consent to participate in this study. Those who were unable to attend the online interview or did not manage patients with chronic conditions were excluded. This was a phenomenal qualitative study; thus, the minimum sample required was six participants (18). Seven eligible HCP were recruited using purposive sampling method for the diversity of disciplines. These specialists were from the field of Internal Medicine, Orthopedics, Rehabilitation Medicine, Oral-maxillofacial Surgery, Otolaryngorhinology, Oncology and Plastic Surgery.

### Study Period

All interviews were conducted via Zoom application between October 2019 and February 2020 with the mean interview duration of 50 min and 10 s.

### Data Collection and Analysis

The interviews were audio recorded, transcribed and thematically analysed using Atlas.ti Cloud software. The coding process of the findings on this study was divided into three stages. In the first stage, ‘open coding’ was conducted where initial codes were assigned line-by-line according to pre-identified themes and new codes were created as they emerged. Subsequently, ‘axial coding’ was done where codes were grouped together and subdivided into pre-determined subdomains or domains. Finally, ‘reorganisation of domains’ was performed where the domains were organised into themes.

## Results

### Patient Generated Health Data Use in Malaysia

PGHD is a relatively new term but its concept is not unheard of. Six out of the seven respondents reported they have never heard of PGHD, previously. However, after given explanation, they confirmed they have been using PGHD in practice without using the term formally. Examples of PGHD used by our respondents are summarised in Table 1. They can be broadly categorised into: i) quantitative data e.g. blood pressure, blood glucose and body weight; ii) qualitative data e.g. PRO questionnaire, quality of life assessment, bladder diary and symptom diary and iii) multimedia data e.g. photos of wound, skin lesion, swelling and deformities.

Most patients were unaware of the concept of PGHD or how it can be used to manage their health. They only started recording PGHD after being prompted by HCP. Even so, some patients might forget to record or bring data to the clinic. According to our respondents, patients’ attitude and adherence towards PGHD vary widely across demographics (Table 2). Younger patients with higher education level or working professional and those living in the cities are more likely to use PGHD. While younger patients were more interested, they often did not require to use PGHD as they were generally healthier than older patients. On the contrary, older patients who are more likely to have medical conditions were less interested to use PGHD. This trend is expected to change in the future when more tech-savvy young people grow older. While some patients found it troublesome to adhere to regular recording of PGHD, some view it as an investment in their health and tend to have better control over their conditions.

**Figure 3:**
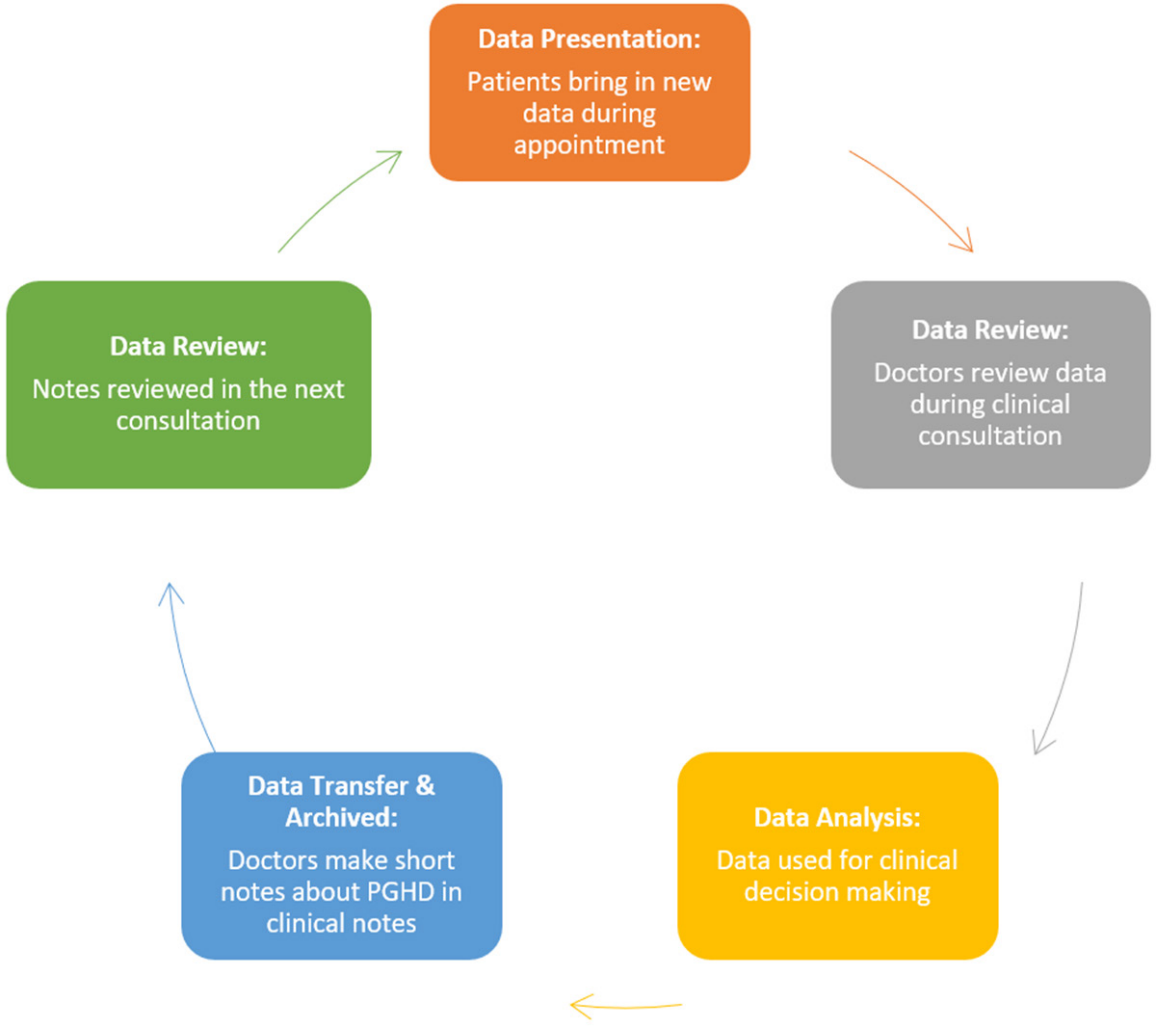
Workflow of PGHD in clinical setting

In general, the process using PGHD can be outlined in Figure 3. Patients were educated by HCP to record their PGHD at home and present them during subsequent clinic visits. PGHD presented is then transcribed and recorded in hospital records and taken into account for clinical decision making.

### Benefits

One of the biggest benefits of PGHD is to collect and manage data more effectively. At present, medical records in HKL are 100% paper-based. All study respondents were favourable towards the usage of electronic PGHD as it can organise and present large data sets in a systematic manner. “If you got proper electronic PGHD, which takes in data and summarise it for you in mean and confidence interval, that’s much easier” (Respondent 1). In addition, electronic data can be transferred and stored easily. Efficient data management can increase the productivity of clinical consultation.

Secondly, PGHD can be used to assist clinical decision making by filling the information gaps about patients’ condition at home between two separate hospital or clinic visits. Most respondents agreed that PGHD can provide more comprehensive clinical information for doctors to devise an individualised management plan. This is especially true in management of chronic conditions such as hypertension, wherein HCP can only capture a snap-shot measurement in the clinic. The latter could be affected by external factors such as anxiety and white coat hypertension. However, PGHD could provide a more consistent and accurate reading of the real picture of patients’ blood pressure control at home. Similarly, asthmatic patients can record their symptoms daily on validated questionnaire such as Asthma Control Questionnaire instead of trying to recall what happened in the past 4 weeks before going into clinic.

Moreover, one study respondent felt that PGHD permits patients to disclose private information voluntarily which is difficult to be disclosed in person. For example, information such as sexual function can be recorded on paper or electronically to present to the doctors.

Last but not least, PGHD can encourage self-efficacy of patients in managing their own health. Our respondents feel that by involving patients in their own healthcare, it empowers them to be more responsible towards their treatment and reinforce them to take care of themselves. Patient who uses PGHD are more likely to be adhering to medications and adapt to lifestyle changes. One study respondent had elaborated on the indirect advantage of PGHD as building better doctor-patient relationship, whereby the patient and physician work together for a common goal of improving clinical outcome.

### Barriers

The most quoted barrier to PGHD use is education level and health literacy of patients. Patients need to be educated to know about their condition, what data to measure and their meaning and what they can do to improve their health. Despite prevalence of smartphones, the majority of respondents had commented on the general lack of awareness of potential use of smartphones in generating and recording PGHD among the public.

Besides, many study respondents had reported that currently most hospitals in Klang Valley do not have ICT infrastructure to support the usage of electronic data. Hopefully, this would change in the future and we can incorporate electronic form of PGHD into EMR.

Another barrier for PGHD usage was the fact that many validated PRO questionnaires and mHealth apps are developed by foreign experts. Thus, some respondents were concerned that patients without good command of English might not be able to use them effectively, especially due to usage of medical terms. They might not be culturally relevant and might not include the presentations of subtropical diseases, which are not commonly seen in the Western world.

Two out of seven respondents mentioned that PGHD has more value in some disciplines such as in internal medicine or community-based service that takes care of patients with chronic conditions than hospital-based surgical specialty that deals with patients with acute presentations. PGHD such as PRO questionnaires are more used in academic settings and is unheard of in private practice.

The respondents had collectively admitted that there was no encouragement from higher authority or incentives in other form for PGHD usage. This could be partly due to lack of awareness and lack of motivation to learn new things. Some doctors might be reluctant to learn and adopt PGHD in practice because they are used to their current way of doing things. HCP have raised concerns about the lack of standardised format of the PGHD used in Malaysia, except the validated PRO questionnaires. Some doctors are worried about the reliability of PGHD as it requires certain techniques to ensure high quality data. For example, the condition in which the patients take their blood pressure reading would affect the accuracy of the reading.

Concern was also raised regarding the possibility of patients confabulating readings in order to please their doctors. One respondent had mentioned that some patients might have false belief that they need to have a set of ‘perfect’ PGHD so as to “make the doctor happy that they (the patient) are compliant” (Respondent 2) or “If they don’t write pretty things on the paper, they are probably not going to get the best chance possible” (Respondent 6).

One respondent had stated that PGHD was inadequate as the sole basis of clinical decision making. “For us to make a correct diagnosis, we can’t just have a look at picture and reading to diagnose, we have to see the patient, that come 30% of the time” (Respondent 2). However, PGHD was never meant to replace in-person consultation and should only serve as a supplement data source to assist clinical decision making. The true value of PGHD might lie in the consistency of monitoring of patients’ well-being while they are at home.

There were conflicting views regarding privacy and confidentiality issue of PGHD. Some respondents believed PGHD might poses extra risk of medico-legal implications as it involves potentially sensitive health information. Other respondents believed that “after the PGHD has been key-in or transcribed into the patient’s folder, that become a hospital property” (Respondent 4).

### Effort Needed to Use Patient Generated Health Data

There are some pre-requisite efforts needed from all parties to use PGHD. Some respondents were concerned about the increased workload to analyse PGHD. It is important to filter out the necessary data as not all of them are clinically significant. Hence, PGHD usage should be led by clinicians to identify the types of data that have clinical value. As one respondent said, “It might be certain data for certain period of time for certain disease, it’s not like we need everything at every time” (Respondent 4).

In order to use PGHD, HCP needs to educate and reinforce patients to record PGHD. While this would require some initial effort at the beginning, it is believed that we can subsequently save time and cost in the long run. In short, all respondents have expressed interest in PGHD and are willing to recommend PGHD to their colleagues and patients.

From the patients’ perspective, they need to learn and be motivated to record PGHD diligently. Some patients might not have time to do so if they are working full-time. Furthermore, some respondents were concerned that PGHD recording could be challenging for certain groups of patients such as elderlies or physically disabled who rely on their carers. In these scenarios, healthcare wearables can come in handy. For example, the latest Apple Watch can measure electrocardiogram, blood oxygen level, pulse rate and steps taken by the users (19). It can alert the patients or the caretakers when something is abnormal and prompt them to seek medical attention (19).

Another big barrier to use PGHD is the cost. In order to generate PGHD, there is an essential cost to buy equipment like the blood pressure machine, glucometer or healthcare wearables. At the minimum, patients need pen and papers to record PGHD at home. For electronic PGHD, they need smartphones and internet plans. Considering the cost, five out of seven respondents felt it can be burdensome for patients from low socioeconomic background and cause healthcare disparity between the well-off patients and those struggling financially.

## Discussion

The lack of awareness about PGHD indicates a deficiency in education and training regarding the recent developments in health informatics across all levels. As a result, there was no prevalent utilisation of PGHD and guidance is also non-existent.

Our respondents stated that most patients were not well versed with their medical conditions and treatments due to low level of health literacy. Without sufficient health literacy, patients cannot utilise PGHD to manage their own health. This needs to be tackled by increasing effort in patient education.

Malaysia, like many other Asian countries, traditionally follow a hierarchical care model (20). The concept of patient-centred care or shared decision making (SDM) is fairly new for the population. Patients often need to be prompted because it was not the cultural norm of the society to actively manage their own health (20). The use of PGHD would encourage shifting the traditional care model towards SDM and patient-centered care.

Asking patients to record symptoms and measurements at home is not a new concept. The main reason why PGHD rose into limelight is due to advancement of electronic data. This requires ICT infrastructure such as internet connectivity, computers and EMR. In terms of PGHD, there are initiatives like Tele-primary Care and Oral Health Clinical Information System featuring self-monitoring modules for patients to report PGHD online for doctors to review (21). ‘The Internet of Things Idea Book’ had outlined the ICT framework for healthcare wearables (22). For example, home monitoring system using healthcare wearable’s paired with MIMOS SoC IoT Processor (Mi-SIP) records patient’s vitals and stores it on Mi-Cloud (22). However, our study did not reflect the fruition of these initiatives as none of the respondents were aware of these developments.

**Figure 4:**
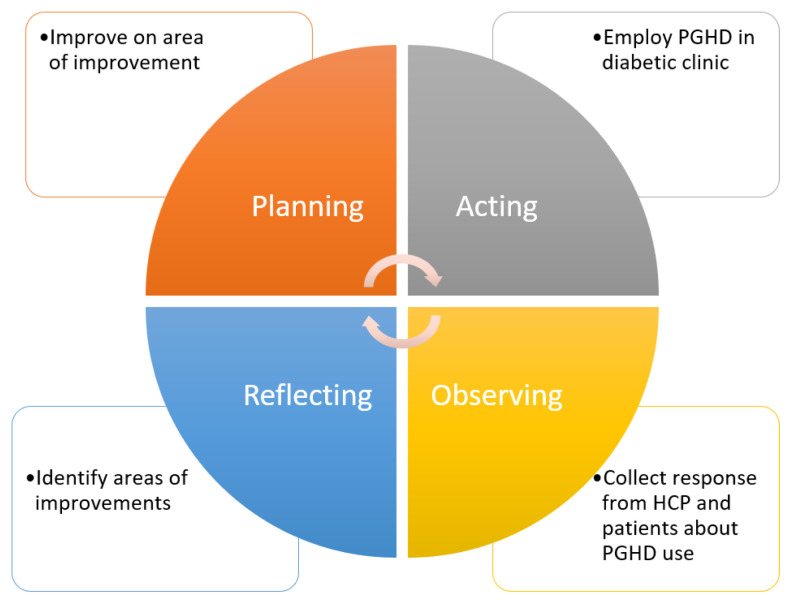
Action research model for PGHD

With advancing technology, healthcare wearables will no doubt become widely accessible. This would make generating PGHD much easier and it would be unwise for the healthcare sector to not utilise this enormous source of valuable data. While the progress is slow, there is a foreseeable future for PGHD to be incorporated into clinical workflow. It is important to recognise the potential of PGHD and promote its usage. By using the results of this study, the below five recommendations to improve PGHD usage in Malaysia have been stated:

- Train HCP and raise public awareness about PGHD
  A campaign promoting PGHD is crucial in raising awareness at all levels of HCP including nurses and allied healthcare professionals. In addition, patients’ information leaflet about PGHD can be produced as a tool for educating patients. Patients need to be educated in order to use PGHD effectively.
- Upgrade hospital ICT infrastructure
  The shortcomings in ICT infrastructure have greatly limited the utilisation of PGHD. Paper data is burdensome to record, transfer, store and navigate as compared to electronic data. Regardless of the intention to use PGHD, upgrading ICT infrastructure is a paramount step to increase the quality of care in the future.
- Develop the market of healthcare wearables and mHealth
  The success of MySejahtera is attributable to the promotion campaign by government and its availability in Malay language. This shows that many Malaysians have the access and ability to use mHealth if tailored to their needs. Local experts should work with HCP on translating or developing apps that feature Malay or multilingual options to suit our local population. For patients from low socioeconomic background, it is possible to arrange social welfare funding to cover the cost to generate PGHD.
- Conduct more research on healthcare wearables and mHealth
  All respondents have emphasised on the importance of evidence-based medicine thereby, showing the data from healthcare wearables and mHealth apps to improve clinical outcome. Evidence is also crucial to convince authorities to allocate more funding. There should be regulatory bodies over-watching the privacy and confidentiality issues of healthcare wearable and mHealth apps. HCP are more willing to use PGHD if there are assurance on the quality and security of data.
- Employ Action Research Model in adopting PGHD
  The pragmatic approach to incorporate PGHD use is by employing action research model (Figure 4), a research activity used to improve practice by employing a cycle of action, evaluation and adaptation based on evidence (23). This involves a trial to implement PGHD in selected healthcare settings on selected cohort of patients and subsequently collect feedback from patients and HCP to evaluate the efficacy and challenges of using electronic PGHD. From these feedbacks, recommendations to improve PGHD usage can be derived and extended to other healthcare settings.

This study provides a unique insight into PGHD in Malaysia. It highlighted the benefits and challenges to use PGHD and derived recommendations to improve its usage. There are several limitations to this study. Firstly, it only included a small number of respondents from HKL; hence, the result is not generalisable to other healthcare settings such as private or university hospitals, general practitioners or rural areas. In addition, this study only included specialists and no other categories of HCP. Further research should explore the views and experience of patients, public and other HCP such as nurses and allied healthcare professionals towards PGHD. Other potential areas of research could focus on data privacy and confidentiality issues and impact of PGHD use in clinical settings.

## Conclusion

This study showed that PGHD is widely accepted among HCP across different specialties and is believed to be the future trend of medicine. At the moment, PGHD in Malaysia is still in immature stage and its benefits are hard to measure due to poor adoption. PGHD usage is not limited for managing chronic conditions but can also be used to monitor general health status and promote healthy lifestyles. With the growing accessibility to healthcare wearables, remote patient monitoring technologies and mHealth, it is strategic to recognise the potential of PGHD and allocate resources towards its promotion in order to catch up with the advancement in healthcare informatics.

## Figures and Tables

**Table 1:** Types of PGHD used in practice

Types of PGHD used		Relevant quotes
Quantitative	Blood pressure Glucose monitoring	“In clinic, a lot of patients bring their own home monitoring for blood glucose level for those who are diabetic and for those who are hypertensive they bring records of their blood pressure.”
	Body weight	“Body weight, for those who are obese, heart failure, end stage renal disease.*”*

Qualitative	PRO questionnaire e.g. Quality of life assessment, the European Organisation for Research and Treatment-Quality of Life questionnaire (EORTC QLQ)-C30	“In terms of oncology, the most commonly used PGHD would probably be their Quality of Life assessment questionnaire. We typically do this in the context of clinical trial as well as any quality of life related research. It’s not part of our routine clinical work to encourage patients but in the concern of a study or a trial then we do it quite diligently.”
	Bladder diary	“Basically it’s a diary, we give a hardcopy, teach them what should they fill in, how much do they pass urine, how much is the urine retained after they CISC (clean intermittent self-catheterization).”
	Pain score	“Normally what we do is pain score, because patients came with severe pain with whatever reason and we do procedure that can help to reduce the pain and at the same time we’d prescribe the painkiller but we will never know how the patients react the different types of painkiller, if we want to know if the pain is more severe in the morning, after meal, before/after sleep then we will ask the patients to record their pain score.”
	Symptoms diary e.g. headaches, fever, sinusitis, recurrent epistaxis and tonsilitis	“They will always come with a paper saying which date they have headaches, sinusitis symptoms, how long it lasted for, what did they do, did they do anything, when did they recover and how long they recovered before the next episode came.”

Multimedia	Photographs of wound and skin condition i.e. swelling and deformities	“They also can come with pictures of their knee swelling, sometimes redness on their skin, deformities and of course, wound as well.” “They take daily photos, they are very conscious of their health and show me the progress, usually during every 2–3 weeks appointment they’ll show me the pictures in between.”

**Table 2:** Demographics of patients and reception to PGHD

More likely to use	Demographics	Less likely to use
Younger	**Age**	Older
Higher	**Education level**	Lower
Professional	**Jobs**	Non-professional
City	**Areas**	Rural

**Relevant quotes**		

“Younger patients are more into digital age, so the exposure is better and better, it’s easier for you to educate them” (Respondent 3)		
“Patient who are not very well educated, or very elderly, tend to be resistant to the whole thing (Respondent 6)		
“If you go to rural areas, you might face abit of challenge, sometimes they don’t even have phones. If it’s city like KL, Pulau Pinang, Johor it’s not a big problem” (Respondent 7)		
